# Dipeptidase Activity and Growth of Heat-Treated Commercial Dairy Starter Culture

**DOI:** 10.1007/s12010-014-1453-6

**Published:** 2014-12-27

**Authors:** Monika Garbowska, Antoni Pluta, Anna Berthold-Pluta

**Affiliations:** 1 Wacław Dąbrowski Institute of Agricultural and Food Biotechnology, Warsaw, Poland; 2 Faculty of Food Sciences, Department of Biotechnology, Microbiology and Food Evaluation, Division of Milk Biotechnology, Warsaw University of Life Sciences-SGGWfsfs, Warsaw, Poland

**Keywords:** Lactic acid bacteria, Proteolysis, Dipeptidase activity, Heat-treated starter culture, Cheese ripening

## Abstract

Growing expectations of consumers of fermented dairy products urge the search for novel solutions that would improve their organoleptic properties and in the case of rennet cheeses-that would also accelerate their ripening process. The aim of this study was to determine the peptidolytic activities and growth of heat-treated commercial culture of lactic acid bacteria. The analyzed culture was characterized by a relatively high peptidolytic activity. The growth of bacterial culture subjected to heat treatment at 50–80 °C for 15 s, 10 and 3 min was delayed by a few or 10–20 h compared to the control culture. Based on the results achieved, it may be concluded that in the production of rennet cheeses, the application of additional, fermentation-impaired starter cultures (via heating for ten or so minutes) may serve to accelerate their ripening and to improve their sensory attributes.

## Introduction

Degradation of casein to peptides, polypeptides and amino acids is of special significance in cheesemaking, because such products of proteolysis as amino acids, alcohols, aldehydes, acids, esters and sulfuric compounds are precursors of specific flavour attributes [24]. A bitter taste resulting from the accumulation of hydrophobic peptides (especially the ones rich in proline) is a quality attribute of Gouda or Cheddar type cheeses [25].

The proteolytic system of lactic acid bacteria consists of three main elements: (1) proteinases bound with a cell wall (CEPs cell-envelope proteinases) that initiate casein degradation to oligopeptides, (2) peptide transport system to cell interior and (3) various intracellular peptidases responsible for degradation of peptides to shorter peptides and amino acids. The first stage of the bacterial degradation of casein involves its hydrolysis by proteinases bound with the cell wall of lactic acid bacteria (LAB). The specificity of extracellular proteinases plays a key role in the synthesis of bitter peptides [1, 21]. Another stage of casein degradation consists in the transport of peptides produced upon CEPs activity to cell interior containing LAB peptidases [5, 10, 18, 22].

Peptidases of LAB cultures participate in bitter peptides degradation and are responsible for organoleptic properties of dairy products [2–4, 15, 17, 26]. However, the ripening of most cheeses requires balanced proteolysis, which plays an important role in flavour profiling, as well as in the prevention of slight bitterness formation in cheeses resulting from the accumulation of bitter polypeptides and peptides [24].

Cheesemaking, including cheese ripening, is a complex, diversified and relatively expensive process. The economic advantages gained by accelerating the process of cheese ripening are obvious. The selection of appropriate basic and additional starter cultures is extremely important at both: cheese production stage and cheese ripening stage. A basic starter culture, which is added to milk mainly to sour it and to assure appropriate ripening, imparts the final and typical sensory properties of a given cheese type. To accelerate the process of cheese ripening, additional fermentation-impaired starter cultures can be used. Protein transformations which proceed during the ripening stage determine in large measure the final and characteristic sensory attributes of cheese. At the first stage of cheese ripening, protein degradation is provoked mainly by rennet enzymes (e.g. rennin). After complete fermentation of lactose, the LABs begin to die gradually and then their impact on protein degradation consists in the activity of proteolytic enzymes released after cell lysis.

The appropriate fermentation activity of a starter influences the proper treatment of a milk curd, the formation of cheese and, to a great extent, also its final properties. This activity cannot, however, be too high because it would cause the over-souring of a cheese bulk and could contribute to the development of cheese defects upon too rapid proteolysis. For this reason, increasing the addition of an active starter is not recommended in cheesemaking. On the other hand, during cheese ripening-especially at the end of this process, the final traits of cheese are profiled by proteolytic enzymes of starter bacteria. One of the still underused methods for cheese ripening acceleration involves the application of an additional (apart from the active starter) heat-treated starter which does not cause over-souring of cheese bulk and contains active proteolytic enzymes that become active during ripening. Although the application of heat-treated LAB cultures brings many advantages, this method is still rarely used in cheesemaking, which may be due to the fact that it alters the final organoleptic traits of cheeses produced with such bacterial cultures and is cost-ineffective. Studies on the use of additional heat-impaired cultures in cheesemaking involved mainly cultures heated at temperatures of 50–72 °C, usually for 10–20 s. Few studies in this range included additional bacterial cultures heated to 65 °C for a few minutes [9]. In this study, the following hypothesis was verified: higher temperature (ranging from 65 to 80 °C) and longer treatment time (10 and 30 min) compared to literature studies (45–70 °C applied most often for tens of seconds) allow to obtain a relatively high peptidolytic activity of selected heat-treated starter culture. The prolonged time (over 10 min) of heat treatment of the additional starter culture would be preferred for practical reasons since the treatment may be carried out under industrial conditions in the starter bulk and does not require as in the case of short times (up to several seconds) the use of extra pasteurizing line in the flow. In view of the above, the aim of this study was to determine the effect of thermal treatment of selected cultures of lactic acid bacteria used in the dairy industry on dipeptidase activity and growth.

## Materials and Methods

### Analytical Material

Dairy starter in the form of multi-strain cultures containing the following bacteria: *Lactococcus lactis* ssp. *cremoris*, *Leuconostoc mesenteroides* ssp. *cremoris*, and *Lactococcus lactis* ssp. *lactis biovar diacetylactis* was analyzed in the study (CHN-19 Chr. Hansen company).

The following eight dipeptides were used in the study: Ala-Ala, Ala-Phe, Phe-Ala, Leu-Gly, Lys-Leu, Tyr-Leu, Tyr-Phe and Ala-Pro (Sigma-Aldrich, Poland) and the following microbiological media were applied: M17 broth (acc. to TERZAGHI, Merck, Poland), phosphate buffer pH 7.0 (Fluka, Poland), and finally, the reagents as follows: ninhydrin (Sigma-Aldrich, Poland), trichloroacetic acid-TCA (POCH, Poland) and ethanol (POCH, Poland).

### Determination of the Activity of Dipeptidases of Lactic Acid Bacteria Culture

The activity of dipeptidases was analyzed with the method described by Moore & Stein [16] and by Khalid et al. [8]. The first step in dipeptidase activity assay included the preparation of a liquid culture of the analyzed starter. To this end, 2 % (2 mL) of the tested starter were added to 100 mL of M17 broth. The culture was incubated at a temperature of 30 °C for 12 h. Afterwards, 10 mL of the culture were transferred and closed to plastic test tubes (*v* = 35 mL) in which it was subject to thermal treatment. The analyzed culture was heat treated at temperatures of 50, 55, 60, 65, 70, 75 and 80 °C for 10, 30 min and for 15 s. The heat treatment proceeded in the following manner: the samples were heated to the applied temperature in a water bath in the presence of a control sample with a thermometer. Once the samples achieved the required temperature for the times of 10 and 30 min respectively, they were transferred to thermostats having appropriate temperatures. In the case of the treatment for 15 s, the samples were heated to the required temperature, then kept in it for 15 s and immediately cooled using a cold water bath containing ice and placed in a fridge having 4 °C. The activity of dipeptidases was also determined in the samples not subjected to thermal treatment.

The samples were centrifuged (5200 g, 4 °C, 25 min, MPW-350R centrifuge) for cell concentration. Then, the cell biomass was double-rinsed with a phosphate buffer pH 7.0 and filled up to the volume of 10 mL. The samples obtained in that way were disintegrated with ultrasounds (Vibra cell, VCX130 disintegrator by Sonics), for 15 min, at 80 % amplitude. Having been disintegrated, the biomass was dissolved in a 0.05 M phosphate buffer (pH 7.0) at the ratio of 1:4 and centrifuged (10,000 *g*, 4 °C, 25 min). After centrifugation, 0.2 mL of the enzymatic extract of cells was sampled and 1.8 mL of substrate (0.05 mM substrate in 0.05 M phosphate buffer pH 7.0) was added. Substrate solutions were always prepared immediately before being used. The enzymatic reaction was run at a temperature of 30 °C and interrupted by adding 2 mL of 24 % TCA, with the mixture left for a few minutes to enable complete reaction. Afterwards, the mixture was filtered through medium-hard filters (POCH, Poland). Next, 1 mL of ninhydrin was added to 1 mL of the sampled filtrate and mixed. The mixture was incubated at a temperature of 100 °C for 10 min, and then immediately cooled. Before spectrophotometric assay, 8 mL of 50 % ethanol were added to the sample. Absorbance was measured with a HELIOSγ spectrophotometer (UVG 130305) at a wavelength of 570 nm. A unit of dipeptidase activity was defined as the change in absorbance by 0.01 within 1 min of the reaction at a temperature of 30 °C expressed per 1 mg of enzymatic protein at a wavelength of 570 nm. The relative activity of dipeptidases (%) was computed compared to the most active enzyme synthesized by the analyzed starter culture.

Samples were also analyzed for protein content, using the Lowry’s method [12].

### Determination of Growth Curves of Heat-Treated Lactic Acid Bacteria Culture

The M17 broth was inoculated with an appropriate amount of the analyzed heat-treated starter culture (2 %). After heat treatment at a temperatures of 50, 55, 60, 65, 70, 75 and 80 °C for 10, 30 min and for 15 s, 300 μL of individual samples were collected into wells of the plate that was next placed in a Bioscreen apparatus (C MBR model) in which the culture was run in. All analyses were performed in triplicate, and three successive assays were carried out. The growth of bacteria proceeded at a temperature of 30 °C for 72 h. Optical density (OD, λ = 420–580 nm) was recorded automatically in a Bioscreen apparatus at 1-h intervals after 10-s shaking of the plate.

### Statistical Analysis

Results obtained were subjected to a statistical analysis with StatGraphicPlus 4.1 software, using the one-way or multi-factor analysis of variance. The significance of differences between mean values was compared with the Tukey’s test at a significance level of α = 0.05.

## Results and Discussion

### Effect of Heat Treatment on Peptidolytic Activity of LAB Culture

For the CHN-19 starter, the enzymatic activity of peptidases degrading the Ala-Ala substrate reached 0.80–2.88 U min^−1^ mg protein^−1^ in the case of the 15-s heat treatment and 5.24 U min^−1^ mg protein^−1^ without heating, 0.00–2.17 U min^−1^ mg protein^−1^ in the case of the 10-min heat treatment and 4.79 U min^−1^ mg protein^−1^ without heating, 1.84–5.28 U min^−1^ mg protein^−1^ in the case of the 30-min heat treatment and 5.50 U min^−1^ mg protein^−1^ without heating (Tables 1, 2 and 3). The highest activity of peptidases of CHN-19 starter against this substrate was determined in the control samples. In the analyzed temperature range, the activity of the CHN-19 starter culture against the Ala-Ala substrate was highly variable. The highest activity of peptidases against this substrate at the 15-s heat treatment was noted at a temperature of 60 °C and reached 2.88 U min^-1^ mg protein^-1^, which constituted ca. 31 % of the relative activity. In turn, at a temperature of 65 °C, the enzymatic activity of peptidases was the lowest and accounted for 0.80 U min^−1^ mg protein^−1^. The range of the relative activity of peptidases of the CHN-19 starter after 15-s, 10-min and 30-min heat treatment was at 8.6–56.3, 0.0–51.3 and 16.7–49.9 %, respectively, for the entire range of temperatures applied, which was indicative of a low degree of hydrolysis of the Ala-Ala substrate.

**Table 1:** Dipeptidase activities of starter CHN-19 heat treated in a temperature range of 50-80 °C for 15 s

Temperature of heat treatment (°C)									
Substrate		C	50	55	60	65	70	75	80
Ala-Ala	Mean	5.24c	2.09ab	1.02ab	2.88b	0.80a	1.94ab	1.78ab	1.24ab
	±SD	1.64	0.35	0.44	0.31	0.59	0.84	0.45	0.44
Ala-Pro	Mean	2.94b	0.00a	0.11a	0.00a	0.00a	0.00a	0.00a	0.00a
	±SD	0.48	0.00	0.09	0.00	0.00	0.00	0.00	0.00
Ala-Phe	Mean	8.95c	9.31c	7.77c	4.66b	1.88a	1.25a	0.00a	0.00a
	±SD	1.19	1.45	0.79	1.10	0.73	0.78	0.00	0.00
Phe-Ala	Mean	6.29c	5.15c	2.90b	1.98ab	0.00a	0.00a	0.00a	0.00a
	±SD	2.03	1.40	0.62	0.26	0.00	0.00	0.00	0.00
Tyr-Phe	Mean	7.90c	8.65c	8.75c	6.29bc	6.22bc	3.65ab	3.28a	1.40a
	±SD	0.49	0.61	1.54	2.33	0.93	0.85	0.78	0.97
Tyr-Leu	Mean	6.48d	4.67cd	5.50cd	3.17bc	1.59ab	0.14a	0.41a	0.06a
	±SD	1.26	1.54	1.61	1.20	1.01	0.11	0.22	0.01
Lys-Leu	Mean	9.28c	5.76b	5.29b	4.55b	1.86a	0.19a	0.31a	0.00a
	±SD	0.84	0.32	1.65	1.07	0.93	0.16	0.12	0.00
Leu-Gly	Mean	7.85e	7.47de	4.75cd	4.47bc	3.52abc	4.71cd	1.82ab	1.09a
	±SD	1.28	1.16	0.20	1.68	1.04	1.45	0.81	0.39

Values (means of five determinations) were expressed as U min^−1^ mg protein^−1^

Means with different letters in line are significantly different (*P* < 0.05)

*C* non-heated sample (control)

**Table 2:** Dipeptidase activities of starter CHN-19 heat treated in a temperature range of 50-80 °C for 10 min

Temperature of heat treatment (°C)									
Substrate		C	50	55	60	65	70	75	80
Ala-Ala	Mean	4.79c	2.09b	2.17b	1.20ab	0.32ab	0.00a	0.59ab	0.00a
	±SD	0.77	0.68	1.07	0.51	0.23	0.00	0.17	0.00
Ala-Pro	Mean	3.11ab	3.82b	1.75a	1.49a	2.74ab	2.52ab	1.85a	1.75a
	±SD	0.42	1.01	0.03	1.03	1.54	0.38	0.42	0.42
Ala-Phe	Mean	8.65e	5.67d	3.56c	5.56d	1.97bc	2.47bc	1.18ab	0.21a
	±SD	0.50	0.68	1.05	1.24	0.53	0.57	0.38	0.16
Phe-Ala	Mean	6.10c	4.12b	3.36b	1.25a	1.36a	1.59a	0.97a	0.91a
	±SD	0.68	0.73	0.47	0.48	0.85	0.50	0.56	0.20
Tyr-Phe	Mean	7.47d	6.66d	5.19cd	3.56bc	1.06ab	0.00a	1.22ab	0.56ab
	±SD	1.57	1.48	1.88	1.58	0.40	0.00	0.45	0.44
Tyr-Leu	Mean	6.19de	4.93cd	6.82e	5.94cde	4.44bc	2.86b	0.64a	0.23a
	±SD	0.45	0.85	0.99	0.89	0.44	0.54	0.47	0.11
Lys-Leu	Mean	9.33d	4.94bc	6.37c	2.80ab	1.56a	1.36a	1.28a	0.86a
	±SD	0.28	1.26	0.75	0.46	0.87	0.43	0.55	0.33
Leu-Gly	Mean	7.66e	5.50d	3.50c	2.32bc	1.57ab	2.44bc	2.57bc	0.71a
	±SD	0.27	0.21	0.85	0.48	0.87	0.68	0.46	0.35

Values (means of five determinations) were expressed as U min^−1^ mg protein^−1^

Means with different letters in line are significantly different (*P* < 0.05)

*C* non-heated sample (control)

**Table 3:** Dipeptidase activities of starter CHN-19 heat treated in a temperature range of 50–80 °C for 30 min

Temperature of heat treatment (°C)									
Substrate		C	50	55	60	65	70	75	80
Ala-Ala	Mean	5.50d	4.92cd	2.45ab	1.84a	2.06a	3.50abc	5.28d	4.06bcd
	±SD	0.85	0.77	0.38	0.25	0.73	0.74	0.97	0.79
Ala-Pro	Mean	3.19b	2.40b	0.67a	0.00a	0.00a	0.00a	0.00a	0.00a
	±SD	0.27	1.11	0.58	0.00	0.00	0.00	0.00	0.00
Ala-Phe	Mean	9.07d	8.77d	9.39d	6.27c	3.80bc	0.02a	1.96ab	0.40a
	±SD	0.85	1.54	0.79	2.07	0.52	0.01	0.36	0.33
Phe-Ala	Mean	6.84d	5.93d	5.86cd	4.81bcd	3.20abc	3.06ab	1.81a	1.81a
	±SD	2.33	0.29	1.39	0.93	0.50	1.09	0.23	0.91
Tyr-Phe	Mean	7.15e	4.45d	3.03c	1.88bc	1.28b	0.00a	0.00a	0.00a
	±SD	0.66	0.83	0.46	0.71	0.22	0.00	0.00	0.00
Tyr-Leu	Mean	6.18d	3.79c	3.39c	1.17b	0.00a	0.00a	0.00a	0.00a
	±SD	0.30	0.56	0.60	0.35	0.00	0.00	0.00	0.00
Lys-Leu	Mean	9.40b	9.17b	11.02b	6.78ab	6.84ab	8.23b	5.71ab	2.40a
	±SD	1.55	3.24	2.35	1.73	2.13	4.07	0.71	0.42
Leu-Gly	Mean	7.42bcd	8.63cd	10.02d	8.85cd	5.06abc	3.76ab	3.47ab	1.29a
	±SD	2.77	2.12	3.33	2.86	0.41	0.73	1.18	0.87

Values (means of five determinations) were expressed as U min^−1^ mg protein^−1^

Means with different letters in line are significantly different (*P* < 0.05)

*C* non-heated sample (control)

In the case of the Ala-Pro substrate, the peptidolytic activity of the CHN-19 starter reached 0.00–0.11 U min^−1^ mg protein^−1^ at 15-s heating and 2.94 U min^−1^ mg protein^−1^ without heating, 1.49–3.82 U min^−1^ mg protein^−1^ at 10-min heating and 3.11 U min^−1^ mg protein^−1^ without heating, 0.00−2.40 U min^−1^ mg protein^−1^ at 30-min heating and 3.19 U min^−1^ mg protein^−1^ without heating (Tables 1, 2 and 3). The highest activity of peptidases of the CHN-19 starter was determined for the sample heated at 50 °C for 10 min (Table 4). Interestingly, in the case of the 10-min heat treatment, the CHN-19 starter culture maintained a similar degree of Ala-Pro hydrolysis in a temperature range of 65–70 °C, i.e., 2.74–2.52 U min^–1^ mg protein^–1^, which corresponded to 28 % relative activity on average, as well as in a temperature range of 75–80 °C, i.e., 1.85–1.75 U min^−1^ mg protein^−1^, which constituted 19 % of relative activity. The ranges of the relative activity of peptidases of the CHN-19 starter heated for 15 s, 10 min and for 30 min reached 0.0–31.5, 16.0–40.9 and 0.0–29.0, respectively, in the whole range of analyzed temperatures. This points to a high activity of peptidases hydrolyzing Ala-Pro upon the longer period of heat treatment (10 min). In the case of the 10-min heat treatment, the enzymes degrading this substrate exhibited high heat resistance in the whole range of applied temperatures. The hydrolysis of substrates containing a proline residue (Xaa-Pro) is attributable to prolidase (PepQ) [11, 28]. Liu et al. 2010 showed that such strains as *L. lactis* ssp. *lactis* IL1403, *L. lactis* ssp. *cremoris* SK11, *L. lactis* ssp. *cremoris* MG1363, and *L. mesenteroides* ATCC 8293 possessed one gene encoding the PepQ enzyme, which is indicative of their capability to hydrolyze the Ala-Pro substrate. The activity of peptidases against Ala-Pro was also demonstrated in samples subjected to heat treatment.

**Table 4:** Statement of heat treatment parameters of CHN-19 culture for which peptidase exhibited maximum activity

Substrate	Temperature of heat treatment	Time of heat treatment	Maximum enzymatic activity (U min^−1^ mg protein^−1^)
Ala-Ala	Lack of treatment	Lack of treatment	5.50
Ala-Pro	50 °C	10 min	3.82
Ala-Phe	55 °C	30 min	9.39
Phe-Ala	Lack of treatment	Lack of treatment	6.84
Tyr-Phe	55 °C	15 s	8.75
Tyr-Leu	55 °C	10 min	6.82
Lys-Leu	55 °C	30 min	11.02
Leu-Gly	55 °C	30 min	10.02

Compared to the above-discussed substrates, the ranges of enzymatic activity of peptidases hydrolyzing Ala-Phe were higher and in the case of the CHN-19 starter heated for 15 s, 10 min and 30 min reached, respectively, 0.00–9.31 U min^−1^ mg protein^−1^ (8.95 U min^−1^ mg protein^−1^ without heating), 0.21–5.67 U min^−1^ mg protein^−1^ (8.65 U min^−1^ mg protein^−1^ without heating) and 0.02–9.39 U min^−1^ mg protein^−1^ (9.07 U min^−1^ mg protein^−1^ without heating) (Tables 1, 2 and 3). It is worthy of note is that the activity of CHN-19 starter peptidases hydrolyzing Ala-Phe was relatively high in a temperature range of 50–65 °C after heating for 15 s. In the samples heated for 10 min, the values of this activity were usually lower in the entire range of analyzed temperatures. The ranges of the relative activity of CHN-19 peptidases degrading the Ala-Phe substrate were at 0.0–100, 2.3–92.7 and 0.2–85.2 % in the samples heated for 15 s, 10 min and 30 min, respectively. The highest peptidolytic activity of the CHN-19 starter against the Ala-Phe substrate was noted in the sample heated for 30 min at a temperature of 55 °C (Table 4).

The activity of peptidases of the analyzed cultures hydrolyzing Phe-Ala, compared to the Ala-Phe substrate, attained lower values which for the CHN-19 starter reached 0.00–5.15 U min^−1^ mg protein^−1^ (6.29 U min^−1^ mg protein^−1^ without heating), 0.91–4.12 U min^−1^ mg protein^−1^ (6.10 U min^−1^ mg protein^−1^ without heating) and 1.81–5.93 U min^−1^ mg protein^−1^ (6.84 U min^−1^ mg protein^−1^ without heating) in the case of the 15-s, 10-min and 30-min heat treatment, respectively. It was observed that along with the prolongation of the heat treatment, the enzymes degrading Phe-Ala substrate were exhibiting higher activity at higher temperatures of the heat treatment, which in a temperature range of 65–80 °C and heating time of 15 s was null, whereas after 30 min and 10 min of heating reached 1.81–3.20 U min^−1^ mg protein^−1^ and 0.91–1.59 U min^−1^ mg protein^−1^, respectively. The relative activity of CHN-19 starter peptidases degrading the Phe-Ala substrate reached 0.0–67.5 % after 15-s, 9.6-65.4 % after 10-min and 16.4-62.1 % after 30-min heat treatment, for the entire range of analyzed temperatures.

The enzymatic activity of the analyzed starter culture against Tyr-Phe attained one of the highest values. The ranges of peptidase activity of the CHN-19 starter were at 1.40–8.75 U min^−1^ mg protein^−1^ (7.90 U min^−1^ mg protein^−1^ without heating), 0.00–6.66 U min^−1^ mg protein^−1^ (7.47 U min^−1^ mg protein^−1^ without heating) and 0.00–4.45 U min^−1^ mg protein^−1^ (7.15 U min^−1^ mg protein^−1^ without heating) for 15-s, 10-min and 30 min heat treatments, respectively (Tables 1, 2 and 3). The highest activity of CHN-19 starter peptidases against Tyr-Phe was demonstrated for the sample heated at a temperature of 55 °C for 15 s (Table 4). For starter culture, the highest activities of peptidases were noted in the samples heated for 15 s. The ranges of the relative activity of CHN-19 starter peptidases degrading the Tyr-Phe substrate reached 15.0–93.9, 0.0–80.1 and 0.0–64.9 % in the samples heated for 15 s, 10 min and 30 min, respectively, in the entire range of experimental temperatures. Values of the relative activity of dipeptidases provided in literature for the Tyr-Phe substrate are characterized by high variability depending on the analyzed microorganism [13, 20].

The peptidolytic activity of the CHN-19 starter against the Tyr-Leu substrate reached 0.06–5.50 U min^−1^ mg protein^−1^ after 15-s heat treatment and 6.48 U min^−1^ mg protein^−1^ without heating, 0.23–6.82 U min^−1^ mg protein^−1^ after 10-min and 6.19 U min^−1^ mg protein^−1^ without heating, 0.00–3.79 U min^−1^ mg protein^−1^ after 30-min and 6.18 U min^−1^ mg protein^−1^ without heating (Tables 1, 2 and 3). The highest peptidolytic activity of the CHN-19 starter against the Tyr-Leu substrate was noted in the sample heated for 10 min at a temperature of 55 °C (Table 4). Values of the relative activity of CHN-19 starter peptidases degrading the Tyr-Leu substrate and heated for 10 and 30 min reached 2.5–66.4 and 0.0–56.1 % respectively, whereas the value of the samples heated for 15 s reached 0.6–69.7 %, in the entire range of experimental temperatures. The activity of peptidases of the CHN-19 starter heat treated at a temperature of 50–70 °C for 10 min against the Tyr-Leu substrate attained the highest values compared to the activity achieved in the same temperature range but upon 15-s treatment. Literature data related to Tyr-Leu hydrolysis are as variable as these reported for the Tyr-Phe substrate and are found to depend upon the analyzed bacterial strain [7, 20].

The activity of CHN-19 starter peptidases against Lys-Leu reached 0.00–5.76 U min^−1^ mg protein^−1^ (9.28 U min^−1^ mg protein^−1^ without heating), 0.86–6.37 U min^−1^ mg protein^−1^ (9.33 U min^−1^ mg protein^−1^ without heating) and 2.40–11.02 U min^−1^ mg protein^−1^ (9.40 U min^−1^ mg protein^−1^ without heating) in the samples heated for 15 s, 10 min and 30 min, respectively (Tables 1, 2 and 3). The highest hydrolyzing activities of enzymes of the CHN-19 starters against Lys-Leu were achieved in the sample heated for 30 min at a temperature of 55 °C (Table 4). The relative activity of peptidases of the 10-min and 30-min heat-treated CHN-19 starter degrading the Lys-Leu substrate ranged from 9.3 to 100 % and from 21.8 to 100 %, respectively. In the case of the 15-s heat treatment of this starter, the relative activity of peptidases was also high and reached 0.0–99.7 %. After 15-s heat treatment of this starter in a temperature range of 70-80 °C, its enzymatic activity was below 3.5 % of the relative activity, whereas after 30-min treatment at 80 °C, it did not exceed 20 %. In the current study, the investigated bacterial cultures were characterized by a high activity of peptidases hydrolyzing the Lys-Leu substrate.

Values of the peptidolytic activity of CHN-19 starter against the Leu-Gly substrate reached 1.09–7.47 U min^−1^ mg protein^−1^ (7.85 U min^−1^ mg protein^−1^ without heating), 0.71 − 5.50 U min^−1^ mg protein^−1^ (7.66 U min^−1^ mg protein^−1^ without heating) and 1.29–10.02 U min^−1^ mg protein^−1^ (7.42 U min^−1^ mg protein^−1^ without heating) after heat treatment for 15 s, 10 min and 30 min, respectively (Tables 1, 2, 3). The highest activity of peptidases of CHN-19 starter degrading the Leu-Gly substrate was assayed in the sample heated for 30 min at a temperature of 55 °C (Table 4). After 15-s heat treatment of the CHN-19 starter, the relative activity of peptidases hydrolyzing Leu-Gly was at 11.7–84.4 %, whereas after 10 and 30-min—at 7.7–82.1 and 11.7–91.0 %, respectively, in the entire range of applied temperatures. The relative activity of peptidases of CHN-19 bacteria against Leu-Gly accounted for ca. 70–80 % in the non-heated samples.

Peptidases of the heat-treated culture were capable of hydrolyzing all analyzed dipeptides. These of the CHN-19 bacteria were characterized by the highest activity against dipeptides that were containing hydrophobic amino acids (leucine, phenylalanine), whose presence was often reported in bitter peptides [29]. This specificity may be advantageous when using an impaired starter in the production of rennet cheeses as a precursor in degradation of bitter peptides and for profiling a desired taste. The lowest activity of CHN-19 starter peptidases against the analyzed dipeptides was determined in the case of the substrate containing proline. Tyrosine-containing dipeptides were hydrolyzed by CHN-19 at a relatively high level (6.82–8.75 U min^−1^ mg protein^−1^). For most enzymes of heat-treated CHN-19 starter culture, the highest activity was found for samples heated at a temperature of 55 °C (Table 4).

**Fig. 1:**
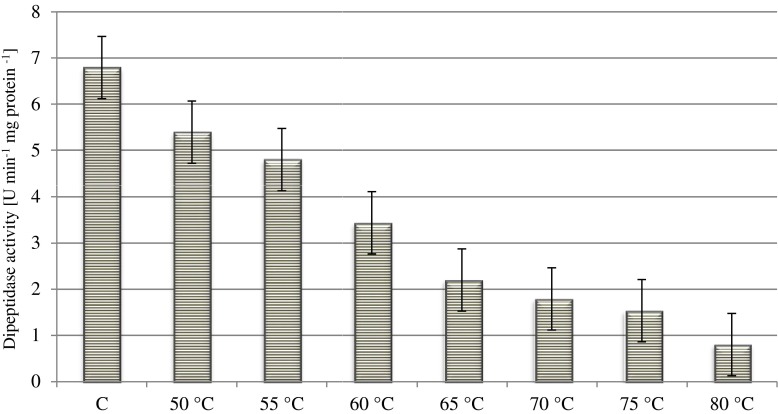
Effect of temperature of heat treatment on the dipeptidase activity of the CHN-19 starter culture (mean and HSD_Tuckey_, *n* = 96) C-non-heated sample (control)

Figure 1 presents the effect of heat treatment temperature on the average peptidolytic activity of the starter culture for all the applied treatment times. The activity of CHN-19 starter peptidases at temperatures of 50, 55, 60, 65, 70, 75 and 80 °C against the control sample accounted for 78, 70, 47, 31, 28, 22 and 14 %, respectively. In this work, a relatively high thermal stability of the dipeptidases of the starter culture bacteria was demonstrated. In some cases, the obtained relative activity of peptidases after heating at 80 °C for 30 min was even 40 %. Moreover, the application of high heat treatment temperature (80 °C) did not completely inhibit peptidase activity of the bacteria of the examined starter.

Tan et al. [27] determined the thermal stability of dipeptidase isolated from *L. helveticus* SBT2171. They found the temperature of 55 °C to be optimal for leucyl-leucine hydrolysis by peptidase. After 30-min incubation of dipeptidases at a temperature of 65 °C they achieved 30 %, whereas upon heating at 75 °C – 20 % of the initial activity. They additionally assayed the thermal stability in a significantly longer treatment time. And so, at a temperature of 50 °C, peptidase was inactivated after 2 h, whereas at 30 °C, it retained 80 % of its activity after 2 h, and after 9 days of incubation, its activity reached even 50 %. In turn, Magboul & McSweeney [14] determined the thermal stability of dipeptidase isolated from *L. curvatus* DPC2024, which was subjected to heat treatment at a temperature of 55, 60, 65 and 70 °C for 0 to 100 min. The enzyme retained 61, 34 and 1 % of its activity after heating for 100 min at a temperature of 55, 60 and 65 °C, respectively. It was relatively stable during 10-min heating at temperatures of 50–60 °C. In turn, dipeptidase originating from *L. casei* subsp. *casei* IFPL731 retained 100 % of its activity after 30-min heating at a temperature of 60 °C [6], whilst Seo et al. [23] demonstrated that after heating at 80 °C for 10 min, the dipeptidase originating from *B. longum* BORI retained 60 % of its initial activity. Results of our study also prove that peptidases of the analyzed heat-treated starter culture retained a relatively high initial activity.

**Fig. 2:**
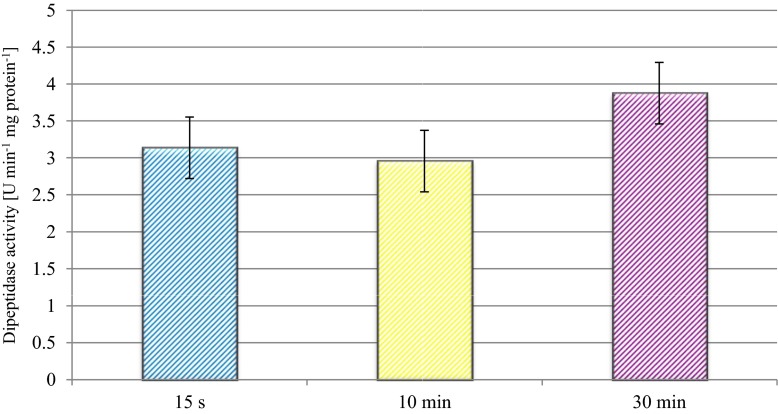
Effect of time of heat treatment on the dipeptidase activity of the CHN-19 starter culture (mean and HSD_Tuckey_, *n* = 256)

After heating in the entire range of experimental temperatures, the average values of peptidase activity of CHN-19 reached 3.14 U min^−1^ mg protein^−1^ in the case of the 15-s heat treatment, 2.99 U min^−1^ mg protein^−1^ in the case of the 10-min heat treatment and 3.38 U min^−1^ mg protein^−1^ in the case of the 30-min heat treatment (Fig. 2). The highest values of peptidase activity were attained after the application of the 30-min heat treatment. These results indicate that the enzymes of CHN-19 starter exhibit high resistance to such a stress factor like temperature and that they even become activated in the course of longer heat treatment.

### Effect of Heat Treatment on the Growth of Culture of LAB

**Fig. 3:**
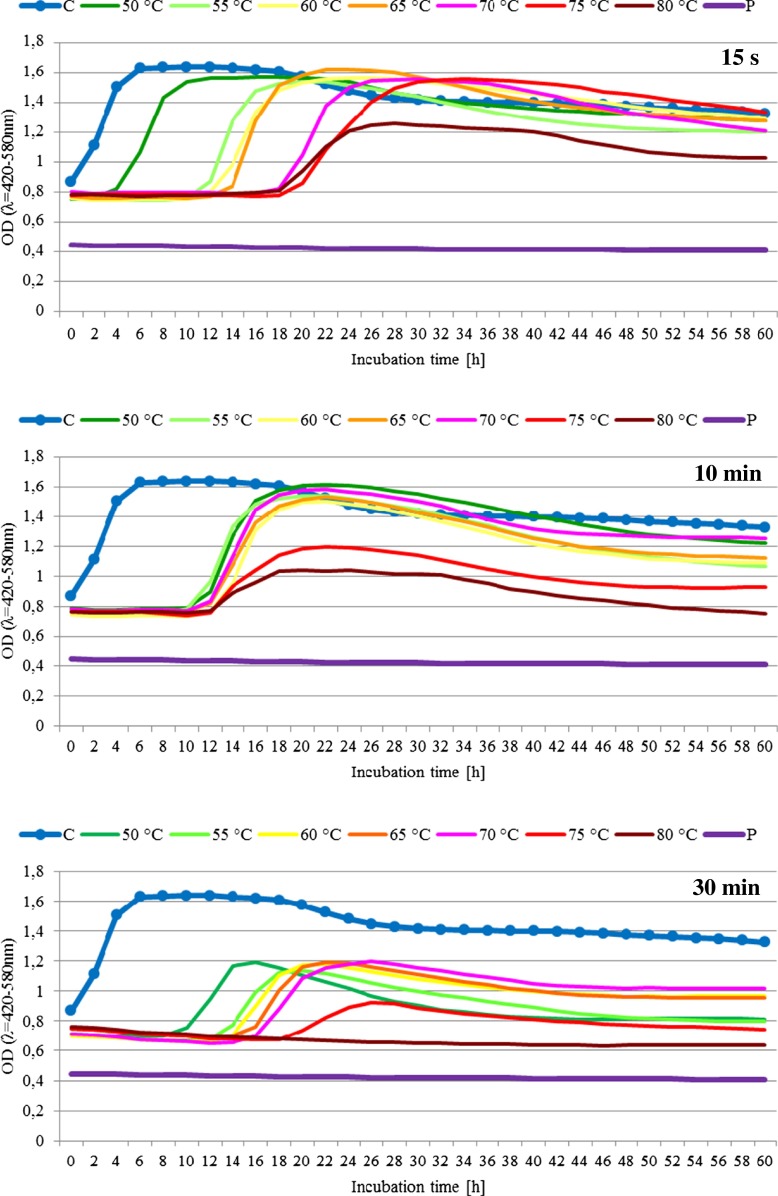
Effect of temperature of heat treatment for 15 s, 10 and 30 min on the growth curve of bacteria of the CHN-19 starter culture. *C* non-heated sample (control), *P* pure culture medium

The study also determined the growth possibility of the examined CHN-19 bacterial culture in the liquid medium after heating in a temperature range of 50–80 °C for 15 s and for 10 and 30 min (Fig. 3).

The growth of CHN-19 starter bacteria was observed after 15-s heating in the entire range of analyzed temperatures. However, their growth was shifted in time compared to the control sample (non-heated). For the samples heated at a temperature of 50, 55, 60, 65, 70, 75, and 80 °C, this shift reached ca. 5, 12, 13, 14, 20, 19, and 19 h, respectively. The bacteria were proliferating to the optical density (OD) of ca. 1.6. Significantly different growth of bacteria was observed after heating the starter for 10 min, as it occurred between the 11th and 14th h of incubation in the case of all heated samples. The samples with bacteria heated at temperatures of 75 and 80 °C reached a lower (ca. 1.4) optical density than the remaining samples (1.6).

In the case of heating CHN-19 starter for 15 s, the temperature of the applied heat treatment affected the beginning of the exponential growth phase and spread it over time. In turn, the 10-min heat treatment caused that the exponential phase was less extended in time. For both heat treatment times of CHN-19 starters, the growth of bacteria heated at a temperature of 80 °C reached a lower value of optical density than in the other temperature variants. Upon both the 15-s and 10-min heat treatment, the surviving bacteria of the CHN-19 starters reached a similar maximum value of optical density.

After earlier heating at a temperature of 80 °C for 30 min, no growth of the starter bacteria CHN-19 was observed when exposed to a temperature of 30 °C for 60 h. Bacteria of the other temperature sample variants after the 30-min heating were able to grow even after 11 h of incubation (i.e., sample heated at a temperature of 50 °C). Moreover, after a long heating time (30 min), the growth of bacteria reached a lower value of optical density (1.2) than in the other time variants.

Prasad et al. [19] analyzed *L. rhamnosus* HN001 strain that was heated at temperatures of 45, 50, 55 °C for 30 min, and determined its growth at a temperature of 37 °C. The earlier heating at a temperature of 45 °C had no effect on the growth of these bacteria, whereas heating at a temperature of 50 °C for 30 min delayed bacteria growth insignificantly and heating at a temperature of 55 °C caused bacteria growth inhibition. However, these authors were determining the growth of bacteria only within 7 h of incubation at a temperature of 37 °C. If such a short period of bacteria incubation had been taken into account in the presented study, it could also be concluded that the growth of bacteria was inhibited as a result of thermal treatment of the starters. In almost all cases, the growth of bacteria of the heat-treated starter CHN-19 occurred after incubation longer than 7 h, i.e. usually after 10-15 h of incubation.

Heat treatment temperature in the range of 65–72 °C destroys 60–80 % of bacteria. In the current study, it was observed that the lactic acid bacteria were capable to proliferate even after heating at a temperature of 80 °C.

The delay of growth of the additional heat-treated culture is positive from the perspective of rennet cheese production, as the additional biomass of bacterial culture – though incapable of growth at the initial stages of cheesemaking but possessing active peptidolytic enzymes - may accelerate the ripening process of cheese and prevent the accumulation of bitter peptides in the cheese bulk that may be synthesized at the first stage of ripening under the influence of rennin or proteinases bound with a cell wall.

## Conclusions

The culture of lactic acid bacteria subjected to heat treatment (50–80 °C for 15 s and for 10 and 30 min) retained their peptidolytic activity at a high level. Peptidases of the attenuated CHN-19 starter were characterized by high substrate specificity to dipeptides containing hydrophobic amino acids, including leucine and phenylalanine. The dipeptidase activity of CHN-19 starter decreased in proportion to the increase of the heating temperature. The heat treatment of the analyzed culture at 50–80 °C was delaying the growth of bacteria for a few to ten or more hours. The study demonstrated also a relatively high thermal stability of intracellular enzymes of bacteria of CHN-19 starter culture heated at temperatures even higher than 70 °C for 15 s. In the applied temperature range, the enzymes from the bacteria of CHN-19 starter having high peptidase activity did not decrease their activity after the 10-min heating, whereas after the 30-min heating, they demonstrated even a higher activity when compared with the result after the 15-s heating. The results obtained are indicative of a relatively high thermal stability of peptidases of the bacteria constituting the analyzed starter culture, which even after high-temperature treatment and long time may be applied in the production of rennet cheeses as a factor both improving sensory properties (degradation of bitter peptides) and accelerating the ripening process.
